# Afucosylated IgG characterizes enveloped viral responses and correlates with COVID-19 severity

**DOI:** 10.1126/science.abc8378

**Published:** 2020-12-23

**Authors:** Mads Delbo Larsen, Erik L. de Graaf, Myrthe E. Sonneveld, H. Rosina Plomp, Jan Nouta, Willianne Hoepel, Hung-Jen Chen, Federica Linty, Remco Visser, Maximilian Brinkhaus, Tonći Šuštić, Steven W. de Taeye, Arthur E. H. Bentlage, Suvi Toivonen, Carolien A. M. Koeleman, Susanna Sainio, Neeltje A. Kootstra, Philip J. M. Brouwer, Chiara Elisabeth Geyer, Ninotska I. L. Derksen, Gertjan Wolbink, Menno de Winther, Rogier W. Sanders, Marit J. van Gils, Sanne de Bruin, Alexander P. J. Vlaar, Amsterdam UMC COVID-19, biobank study group, Theo Rispens, Jeroen den Dunnen, Hans L. Zaaijer, Manfred Wuhrer, C. Ellen van der Schoot, Gestur Vidarsson

**Affiliations:** 1 Department of Experimental Immunohematology, Sanquin Research, Amsterdam, Netherlands.; 2 Landsteiner Laboratory, Amsterdam UMC, University of Amsterdam, Amsterdam, Netherlands.; 3 Center for Proteomics and Metabolomics, Leiden University Medical Center, Leiden, Netherlands.; 4 Department of Rheumatology and Clinical Immunology, Amsterdam UMC, Amsterdam Rheumatology and Immunology Center, Amsterdam, Netherlands.; 5 Department of Experimental Immunology, Amsterdam UMC, University of Amsterdam, Amsterdam, Netherlands.; 6 Department of Medical Biochemistry, Experimental Vascular Biology, Amsterdam UMC, University of Amsterdam, Amsterdam, Netherlands.; 7 Department of Cardiovascular Sciences, Amsterdam Infection and Immunity Institute, University of Amsterdam, Amsterdam, Netherlands.; 8 Finnish Red Cross Blood Service, Helsinki, Finland.; 9 Department of Medical Microbiology, Amsterdam UMC, Amsterdam Infection and Immunity Institute, University of Amsterdam, Amsterdam, Netherlands.; 10 Department of Immunopathology, Sanquin Research, Amsterdam, Netherlands.; 11 Landsteiner Laboratory, Amsterdam UMC, University of Amsterdam, Amsterdam, Netherlands.; 12 Amsterdam Rheumatology and Immunology Center, Reade, Amsterdam, Netherlands.; 13 Weill Medical College, Cornell University, New York, USA.; 14 Department of Intensive Care Medicine, Amsterdam UMC (Location AMC), University of Amsterdam, Amsterdam, Netherlands.; 15 Department of Blood-borne Infections, Sanquin, Amsterdam, Netherlands.

## Abstract

Immunoglobulin G (IgG) antibodies are crucial for protection against invading pathogens. A highly conserved N-linked glycan within the IgG-Fc tail, which is essential for IgG function, shows variable composition in humans. Afucosylated IgG variants are already used in anticancer therapeutic antibodies for their increased activity through Fc receptors (FcγRIIIa). Here, we report that afucosylated IgG (approximately 6% of total IgG in humans) are specifically formed against enveloped viruses but generally not against other antigens. This mediates stronger FcγRIIIa responses but also amplifies brewing cytokine storms and immune-mediated pathologies. Critically ill COVID-19 patients, but not those with mild symptoms, had high concentrations of afucosylated IgG antibodies against severe acute respiratory syndrome coronavirus 2 (SARS-CoV-2), amplifying proinflammatory cytokine release and acute phase responses. Thus, antibody glycosylation plays a critical role in immune responses to enveloped viruses, including COVID-19.

## A single sugar makes all the difference

Antibodies are divided into several classes based on their nonvariable tail (Fc) domains. These regions interact with disparate immune cell receptors and complement proteins to help instruct distinct immune responses. The Fc domain of immunoglobulin G (IgG) antibodies contains a conserved N-linked glycan at position 297. However, the particular glycan used at this position is highly variable. IgG lacking core fucosylation at this position initiates enhanced antibody-dependent cellular cytotoxicity by increased affinity to the Fc receptor FcRIIIa. Larsen *et al.* report that COVID-19 patients with severe symptoms have increased levels of anti–severe acute respiratory syndrome coronavirus 2 (SARS-CoV-2) IgG afucosylation compared with patients with mild disease. These findings suggest that treatment of COVID-19 patients with fucosylated anti–SARS-CoV-2 antibodies may circumvent pathologies associated with severe COVID-19.

*Science*, this issue p. eabc8378

## Abstract (structured)

**INTRODUCTION:**

Antibody function is often considered static and mostly determined by isotype and subclass. The conserved N-linked glycan at position 297 in the Fc domain of immunoglobulin G (IgG) is essential for an antibody’s effector functions. Moreover, this glycan is highly variable and functionally relevant, especially for the core fucose moiety. IgG lacking core fucosylation (afucosylated IgG) causes increased antibody-dependent cellular cytotoxicity (ADCC) through highly increased IgG-Fc receptor IIIa (FcγRIIIa) affinity. Despite constant levels of total plasma IgG-Fc fucosylation above 90%, specific IgG responses with low core fucosylation have been sporadically reported. These are directed against alloantigens on blood cells and glycoproteins of HIV and dengue virus. In this study, we investigated the induction of afucosylated IgG to various antigens and delineated its dynamics and proinflammatory potential in COVID-19.

**RATIONALE:**

Afucosylated IgG responses have only been found in various alloimmune responses against cellular blood groups and two enveloped viruses. Therefore, we tested the hypothesis that foreign surface–exposed, membrane-embedded proteins induce a specific B cell response that results in afucosylated IgG. We compared immune responses to natural infections by enveloped viruses and nonenveloped viruses, protein subunit vaccination, and live attenuated virus vaccinations. We also assessed the relation to the clinical outcome of such a response in COVID-19.

**RESULTS:**

Analogous to blood cell alloantigens, the response to all enveloped viruses showed clear signatures of afucosylation of the antigen-specific IgG. By contrast, IgG against the nonenveloped virus, parvovirus B19, were highly fucosylated. The extent of afucosylated IgG responses varied, both between individuals and between antigens. The viral context was essential to induce afucosylated IgG because induction did not occur after subunit vaccination against hepatitis B virus. However, afucosylated IgG responses were found in response to attenuated viruses. Severe acute respiratory syndrome coronavirus 2 (SARS-CoV-2)–specific afucosylated IgG were also found in critically ill COVID-19 patients but not in individuals with mild symptoms. Over the 2 weeks after seroconversion, the amount of fucosylated anti–SARS-CoV-2 IgG increased markedly, in most reaching relative levels similar to those found in total IgG. Afucosylated IgG promoted interleukin-6 (IL-6) release in macrophages cultured in vitro, which is in line with an observed association of SARS-CoV-2–specific IgG afucosylation with IL-6 and C-reactive protein (CRP) in these patients.

**CONCLUSION:**

This work suggests that providing foreign B cell antigens in the context of host cells may be required to trigger an afucosylated IgG immune response. The strength of this response is highly variable for different antigens and between individuals. An afucosylated IgG response is a potent immune response, honed for the destruction of target cells by FcγRIII-expressing natural killer (NK) and myeloid cells. This may sometimes be desirable—for example, against HIV—and can be achieved in vaccines by providing the target as a surface protein, as is the case with attenuated viral vaccines or mRNA vaccines. However, for SARS-CoV-2, this afucosylated IgG response may promote the exacerbation of COVID-19 under conditions with high viral loads at the time of seroconversion.

## Teaser

Patients with severe COVID-19 show heightened afucosylated antiviral antibody responses.

## Graphical abstract

**Figure:**
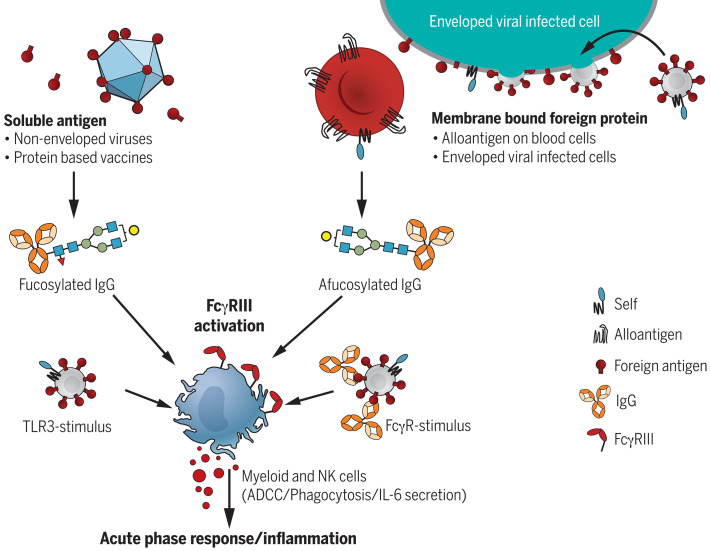
Afucosylated IgG response requires membrane context and results in strong FcγRIII-mediated activity. Only membrane association on host cells endows foreign antigens to trigger the B cell receptor in the context of other self receptors, leading to an afucosylated IgG response. The elevated FcγRIII binding and activity of afucosylated IgG can in some cases be protective, but for SARS-CoV-2, this triggers excessive inflammation during a natural infection.

Antibody function has long been considered static and mostly determined by their isotype and subclass. The presence of a conserved N-linked glycan at position 297 in the Fc domain of immunoglobulin G (IgG) is essential for its effector functions (*1*–*3*). Moreover, the composition of this glycan is highly variable, which has functional consequences (*2*–*4*). This is especially true for the core fucose attached to the Fc glycan. The discovery that IgG variants without core fucosylation cause elevated antibody-dependent cellular cytotoxicity (ADCC), through increased IgG-Fc receptor IIIa (FcγRIIIa) affinity (*5*, *6*), has resulted in next-generation glyco-engineered monoclonal antibodies (mAbs) that lack core fucosylation for targeting tumors (*7*).

Generally, changes in the Fc glycans are associated with age, sex, and autoimmune diseases and are most pronounced for IgG-Fc galactosylation, which decreases steadily with advancing age. After a marked elevation in young women, IgG-Fc galactosylation decreases during menopause to the levels seen in men (*8*). IgG-Fc fucosylation is more stable, decreasing slightly from birth to ~94% at adulthood (*9*), after which it remains fairly constant, albeit with a minor reduction throughout life (*8*, *10*).

Despite the apparent constant level of Fc fucosylation during adulthood, alloantibodies against red blood cells (RBCs) and platelets show low IgG-Fc fucosylation in most patients, even down to 10% in several cases (*11*–*13*). By contrast, overall serum IgG-Fc fucosylation is consistently high. Moreover, lowered IgG-Fc fucosylation is one of the factors that determine disease severity in pregnancy-associated alloimmunizations, resulting in excessive thrombocytopenia and RBC destruction when targeted by afucosylated antibodies (*12*–*14*). In addition to the specific afucosylated IgG response against platelets and RBC antigens, this response has only been identified against human immunodeficiency virus (HIV) and dengue virus (*15*, *16*). Low core fucosylation of anti-HIV antibodies has been suggested to be a feature of elite controllers of infection, whereas for dengue, it has been associated with enhanced pathology owing to excessive FcγRIIIa activation (*15*, *16*). The mechanisms that control IgG core fucosylation remain unclear, however.

Similar afucosylated IgG are found in various alloimmune responses (*11*–*13*, *17*), HIV (*16*), and dengue (*15*), which are all directed against surface-exposed, membrane-embedded proteins. Therefore, we analyzed IgG glycosylation in antihuman platelet responses and in natural infections by enveloped viruses, including HIV, cytomegalovirus (CMV), measles virus, mumps virus, hepatitis B virus (HBV), and severe acute respiratory syndrome coronavirus 2 (SARS-CoV-2). We also assessed responses to a nonenveloped virus (parvovirus B19), vaccination with a HBV-protein subunit, and live attenuated enveloped viruses to test whether the antigen context was an important determinant for IgG-Fc glycosylation.

## Afucosylated IgG is formed against enveloped viruses

**Fig. 1:**
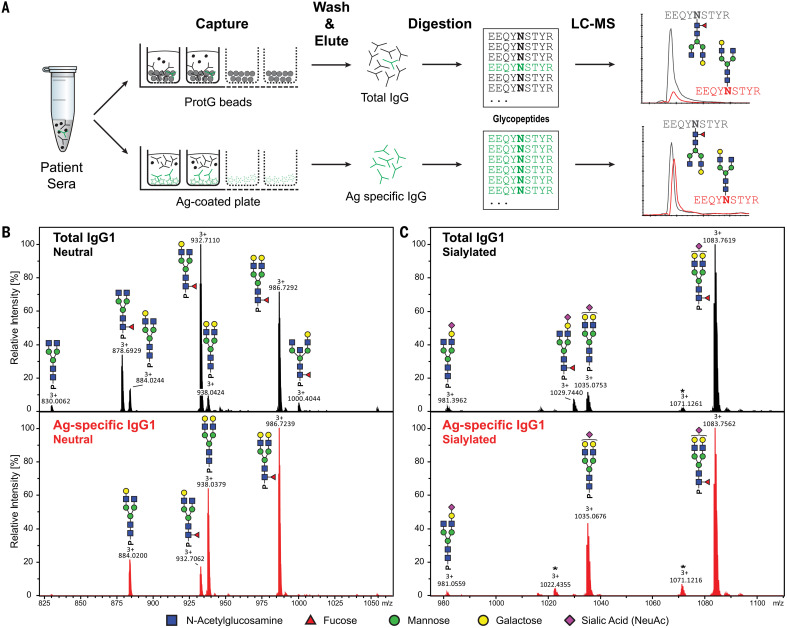
Flowchart of antibody-specific IgG1 glycosylation analysis by use of mass spectrometry. (**A**) Antibodies were captured from sera by using Protein G beads and antigen-coated 96-well plates, resulting in total and antigen-specific IgG fractions, respectively. Thereafter, isolated IgG were digested with trypsin, and the resulting glycopeptides were analyzed by means of nano–LC-MS. (**B** and **C**) Representative mass spectra of glycopeptides encompassing the Fc glycosylation site Asn^297^. (B) Neutral and (C) sialylated IgG1 glycopeptides are shown from a single patient’s total (top, black) and antigen-specific (bottom, red) IgG1 fraction. Asterisks indicate non-Fc glycopeptides.

**Fig. 2:**
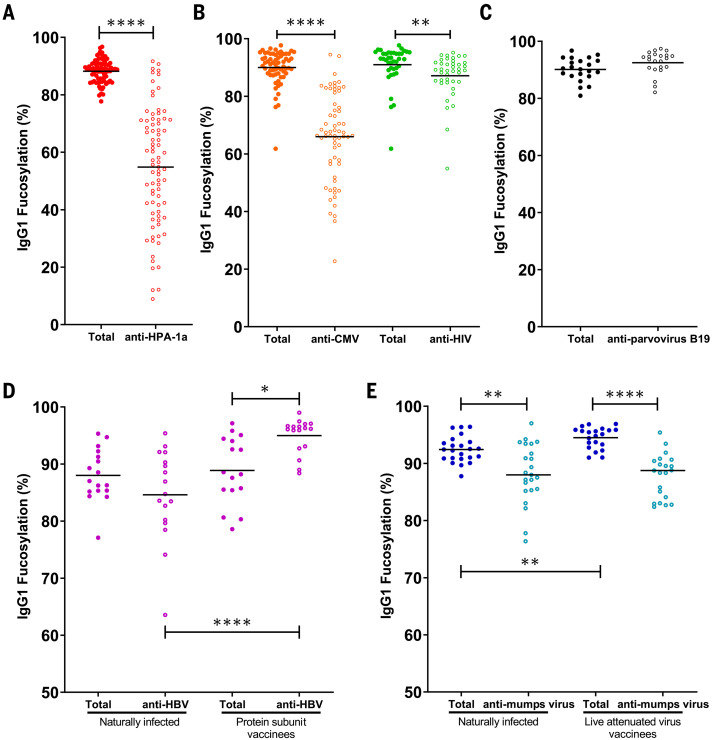
Foreign membrane protein antigens, such as envelope proteins of (attenuated) enveloped viruses or alloantigens, can trigger afucosylated IgG responses. (**A** to **E**) IgG1-Fc fucosylation levels of total (solid circles) and antigen-specific (open circles) antibodies are shown for each differently color-coded group of antigens: (A) alloantigen HPA-1a; (B) viral envelope antigens from CMV and HIV; (C) nonenveloped viral antigens from parvovirus B19; (D) HBsAg, in individuals (left) naturally infected with HBV or (right) vaccinated with recombinant soluble HBsAg; and (E) mumps virus antigens in individuals (left) naturally infected with mumps virus or (right) vaccinated with live attenuated mumps virus. Each circle represents a biological replicate [(A) *n* = 80 for anti-HPA-1a, (B) *n* = 65 for CMV and *n* = 40 for HIV, (C) *n* = 22 for B19, (D) *n* = 17 for naturally infected individuals for HBV and *n* = 17 for HBV vaccinated individuals, and (E) *n* = 24 naturally infected individuals for mumps virus and *n* = 21 for mumps vaccinated individuals] of a representative LC-MS run (examples of technical replicates are provided in fig. S1C). Statistical analyses were performed as paired *t* tests for (A), (B), and (C), and a mixed-model two-way ANOVA with Bonferroni correction of post hoc *t* tests for comparing Fc fucosylation between groups was performed for (D) and (E). Only statistically significant differences are shown. *P < 0.05, **P < 0.01, and ****P < 0.0001.

IgG-Fc glycosylation of affinity-purified total and antigen-specific antibodies were made possible with tandem liquid chromatography–mass spectrometry (LC-MS) (Fig. 1 and fig. S1) (*12*, *17*, *18*). Fc fucosylation of antigen-specific antibodies against the alloantigen human platelet antigen 1a (HPA-1a) were substantially reduced (Fig. 2A) (*14*), which is similar to previous findings for other alloantigens (*12*, *17*). Analogous to platelet and RBC alloantigens (*11*–*13*, *17*), the response to the enveloped viruses CMV and HIV also showed significant afucosylation of the antigen-specific IgG (Fig. 2B). By contrast, IgG against the nonenveloped virus parvovirus B19 were fucosylated (Fig. 2C). The total IgG showed high fucosylation levels throughout (Fig. 2, A to C), reaffirming previous findings that the majority of IgG responses result in fucosylated IgG (*12*, *18*, *19*). The extent of afucosylated IgG responses to the enveloped viruses was highly variable, both between individuals and between the types of antigen, which is similar to observations of immune responses to different RBC alloantigens (*17*). Afucosylation was particularly strong for CMV and less pronounced for HIV (Fig. 2B), confirming previous observations in HIV (*16*). Afucosylated IgG responses were often accompanied by increased galactosylation (fig. S2).

To test whether some individuals had a greater intrinsic capacity to generate an afucosylated IgG response than others, we compared IgG1-Fc fucosylation levels against two different antigens within the same individual. No correlation was observed when comparing the level of afucosylation between two different antigens within the same individual, neither for anti-HPA-1a and anti-CMV (fig. S3A) nor for anti-HIV and anti-CMV antibodies (fig. S3B). Thus, the level of afucosylation is not predetermined by general host factors such as genetics but is rather stochastic or multifactorial, with the specific triggers remaining obscure.

## Afucosylated IgG is generated against attenuated enveloped viral vaccines

To further investigate the immunological context by which potent afucosylated IgG is formed, we compared immune responses to identical viral antigens in different contexts. First, we compared hepatitis B surface antigen (HBsAg)–specific antibody glycosylation in humans naturally infected with HBV or vaccinated with the recombinant HBsAg protein (Fig. 2D). Total IgG1-Fc fucosylation levels were similar for the two groups, whereas anti-HBsAg IgG1-Fc fucosylation was elevated in individuals vaccinated with the HBsAg protein when compared with either total IgG- or antigen-specific IgG-Fc fucosylation of the naturally infected group (Fig. 2D). Thus, HBsAg-specific antibodies in individuals who cleared a natural infection show lowered Fc fucosylation compared with that in individuals who received protein subunit vaccination. This strongly suggests that a specific context for the antigenic stimulus is required for afucosylated IgG responses.

We then compared antiviral IgG responses against mumps and measles viruses formed after a natural infection or vaccination with live attenuated viruses. Unlike the HBV protein subunit vaccine, both live attenuated vaccines showed a similar antigen-specific Fc fucosylation compared with their natural infection counterpart (Fig. 2E and fig. S4). The tendency to generate afucosylated IgG was weak for measles, whereas the mumps response showed clear signs of afucosylation by either route of immunization (Fig. 2E and figs. S4 and S5).

## Afucosylated IgG is found in critically ill COVID-19 patients

**Fig. 3:**
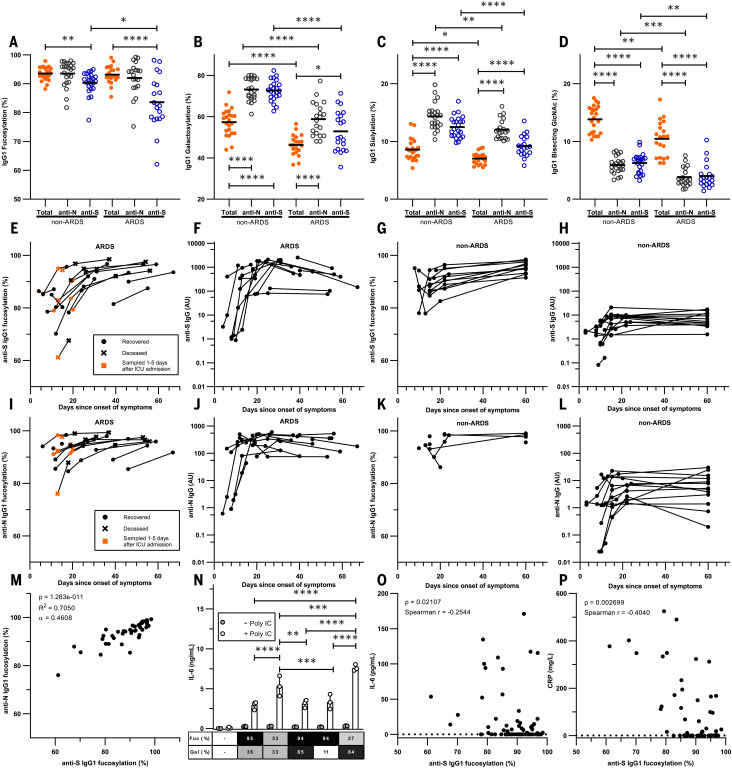
Fc fucosylation levels of anti-S IgG1 are significantly decreased in critically ill COVID-19 patients. (**A**) Fc fucosylation, (**B**) galactosylation, (**C**) sialylation, and (**D**) bisection degree of anti-S, anti-N, and total IgG1 from ARDS patients and non-ARDS donors clearing the infection asymptomatically or with mild symptoms from the initial screen. (**E** to **L**) Longitudinal IgG1-Fc fucosylation and IgG quantity for [(E) to (H)] anti-S and [(I) to (L)] anti-N in [(E), (F), (I), and (J)] ARDS patients and [(G), (H), (K), and (L)] non-ARDS cases. (**M**) Correlation between anti-N and anti-S IgG1-Fc fucosylation. (**N**) Representative examples of IL-6 release from macrophages triggered by FcγR through stimulation with glycoengineered IgG complexes with or without polyinosinic:polycytidylic acid [poly(I:C)]. (**O**) Correlation between plasma IL-6 concentrations and degrees of anti-S IgG1-Fc fucosylation. (**P**) Correlation between plasma CRP concentrations and degrees of anti-S IgG1-Fc fucosylation. Each circle represents a biological replicate: *n* = 20 for ARDS, *n* = 23 for non-ARDS [(A) to (D)], *n* = 17 and *n* = 14 for longitudinal ARDS and non-ARDS, respectively [(E) to (L)]. Examples of technical replicates for LC-MS data are shown in fig. S1C. [(F), (H), (J), and (L)] IgG data are representative ELISA values calibrated against a standard pool from two technical experiments. For all available paired data used in (M), *n* = 40. (N) IL-6 production by macrophages was measured with ELISA, with each dot (*n* = 3) representing a technical replicate. All six biological replicates are shown in fig. S13. CRP and IL-6 numbers were obtained from clinical parameters and IL-6 data by Meso Scale Discovery, using all available paired data [(O) *n* = 82 and (P) *n* = 53]. Statistical analyses were performed as a mixed-model two-way ANOVA with Bonferroni correction of post hoc *t* tests for comparing glycosylation traits and cytokine secretion between groups. Spearman’s correlations were performed in (O) and (P). To test the correlation between Fc fucosylation levels for anti-S and anti-N, a Pearson’s correlation was performed. Only statistically significant differences are shown. *P < 0.05, **P < 0.01, ***P < 0.001, ****P < 0.0001.

We then tested whether this type of response also plays a role in patients with COVID-19. Symptoms of COVID-19 are highly diverse, ranging from asymptomatic or mild self-limiting infection to a severe airway inflammation that leads to acute respiratory distress syndrome (ARDS), often with a fatal outcome (*20*, *21*). Both extreme trajectories follow similar initial responses: Patients have approximately a week of relatively mild symptoms, followed by a second wave that either resolves the disease or leads to a highly aggravated life-threatening phenotype (*20*, *21*). Both the timing of either response type and the differential clinical outcome suggested different routes taken by the immune system to combat the disease. So far, no clear evidence has emerged that can distinguish between these two hypothetical immunological paths. In accordance with our hypothesis and responses observed against other enveloped viruses, anti-S IgG responses against SARS-CoV-2 spike protein (S), which is expressed on the cell surface and the viral envelope, were strongly skewed toward low levels of core fucosylation. By contrast, responses against the nucleocapsid protein (N), which is not expressed on the cell surface or viral envelope, were characterized by high levels of fucosylation (Fig. 3A). The IgG response appeared to be highly specific for SARS-CoV-2 because there was very weak or absent reactivity to SARS-CoV-2 antigens in pre-outbreak samples, even to the more conserved N antigen (fig. S6) (*22*). The anti-S IgG1 responses of patients with ARDS recently (<5 days) hospitalized in intensive care units (ICUs) were significantly less fucosylated than in convalescent plasma donors consisting of individuals who were asymptomatic or had relative mild symptoms (non-ARDS) (Fig. 3A).

These decreased levels of Fc fucosylation of anti-S IgG were not a result of inflammation because total IgG-Fc fucosylation levels were similar between the two groups and to what has been reported in the general population (~94%) (*12*, *18*). In addition, IgG1-Fc galactosylation and sialylation of both anti-S and anti-N responses (Fig. 3, B and C) were significantly increased compared with total IgG, which is consistent with reports describing increased Fc galactosylation and sialylation in active or recent immunization (*18*, *23*). Total IgG1-Fc galactosylation and sialylation levels were significantly lowered in the ARDS patients, which was perhaps a reflection of a slight age difference between these two groups [non-ARDS donors median age (IQR) 49 (40 to 55) years versus ARDS patients 60 (55–63) years (tables S1 and S2)]. Both Fc galactosylation and sialylation decrease with age (*9*, *19*). Increased galactosylation and sialylation of antigen-specific IgG1-Fc increases complement activity by approximately three- to fourfold. Fc galactosylation further enhances affinity of afucosylated IgG to FcγRIII by approximately twofold (*24*). Last, although Fc bisection was significantly lowered in both anti-N and anti-S responses (Fig. 3D), the biological and clinical relevance of this is limited because IgG-Fc bisection affects neither Fc receptor nor complement activity (*24*). Further, accumulating evidence strongly suggests that the primary and major biologically relevant change in IgG-Fc glycosylation is the lack of core fucose. Afucosylated IgG have a 20- to 40-fold increase in affinity to FcγRIIIa, which is often accompanied by an absolute change from no cellular response to strong phagocytic and ADCC responses upon afucosylation (*5*, *15*, *24*, *25*). The lowered Fc fucosylation in the anti-S responses of the ARDS patients suggests a pathological role through FcγRIIIa, similar to what has previously been proposed for dengue (*15*). In dengue, non-neutralizing antibodies that were formed to previous infections of other dengue serotypes also tend to have low amounts of core-fucosylated IgG. Because they are incapable of preventing infection, they lead to aggravated dengue hemorrhagic fever because of FcγRIIIa-mediated overreactions by immune cells (*15*).

## Fucosylation levels of anti-S are lowest at seroconversion

ARDS patients were sampled within 1 week after ICU admission, and non-ARDS patients were convalescent nonhospitalized individuals. In order to eliminate any possible sampling bias in the observed IgG-Fc glycosylation patterns over time, we also analyzed longitudinal samples from both groups (*26*). Alloantibody Fc fucosylation to platelets and RBC antigens is stable for at least a decade with or without a natural booster through pregnancies (*12*, *14*) or blood transfusion (*27*). This also held true for anti-CMV and anti-HIV responses (fig. S7). By contrast, changes in all glycosylation traits were already observed for SARS-CoV-2 during the first week after ICU admission (Fig. 3, E to L, and figs. S8 and S9). These observed changes in Fc galactosylation (fig. S8) were in line with previous reports that recent immunizations are accompanied with a transient rise in antigen-specific IgG-Fc galactosylation and sialylation (*18*, *23*). After seroconversion, all ARDS patients initially showed low levels of anti-S IgG fucosylation compared with non-ARDS patients. Fucosylation levels rose over time in ARDS patients, reaching levels comparable with those of the non-ARDS cohort (Fig. 3, E and G). The increases in fucose levels were associated with simultaneous rises in IgG levels (Fig. 3, E to H, and fig. S10, A and B), which were much less pronounced in the non-ARDS cohort. Similar kinetics were observed for anti-N IgG numbers (Fig. 3, J and L). Reduced levels of anti-N Fc fucosylation were also present in the ARDS group, although to a lesser degree than for anti-S (Fig. 3, I and K, and fig. S11). This unexpected reduction in the fucosylation of anti-N IgG seen in the ARDS cohort may have been the result of classical bystander effects (*28*). Namely, B cells proliferating in the same lymphoid organs receive similar environmental cues from antigen-presenting cells and T cells. The IgG1-Fc fucosylation of anti-S and anti-N correlated significantly (Fig. 3M) with higher levels of afucosylation for anti-S (*P* < 0.0001). Significant correlations were also observed for other glycosylation traits, with similar skewing for both anti-S and anti-N IgG (fig. S12). These elevations in antigen-specific IgG1-Fc galactosylation and sialylation agreed with earlier reports that have suggested that these are general features of newly formed ongoing immune responses (*18*, *23*). Total IgG1-Fc fucosylation remained stable throughout the observation period (figs. S8J and S9J).

## Afucosylated IgG contributes to inflammation in COVID-19

**Fig. 4:**
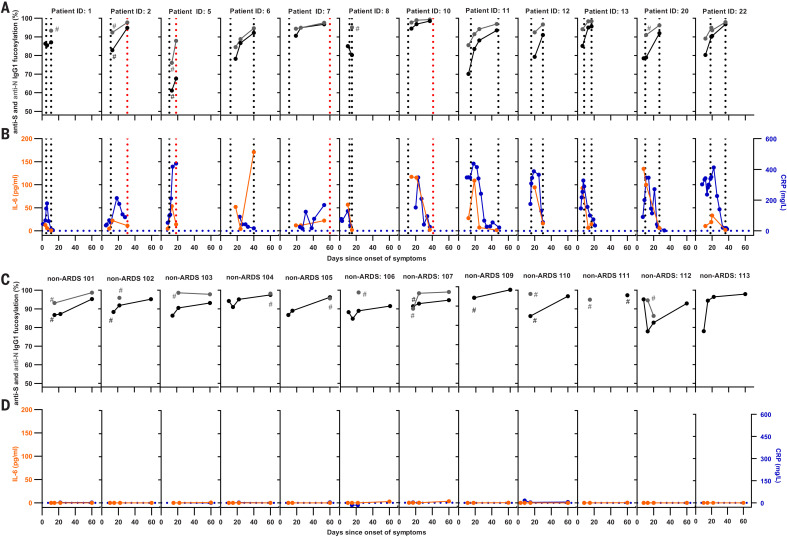
Longitudinal changes of anti–SARS-CoV-2 IgG1 Fc fucosylation, CRP, and IL-6. (**A** and **C**) Anti-S IgG fucosylation and anti-N IgG fucosylation and (**B** and **D**) IL-6 and CRP amounts in [(A) and (B)] an ARDS cohort and [(C) and (D)] a non-ARDS cohort. (A) to (D) represent longitudinal biological replicates of [(A) and (C)] a LC-MS run (examples of technical replicates are available in fig. S1C), CRP obtained from clinical parameters, and [(B) and (D)] IL-6 data by using a validated Meso Scale Discovery assay (*n* = 12 for ARDS and *n* = 14 for non-ARDS, with 2 to 16 longitudinal replicates per patient as indicated). Additional non-ARDS samples are provided in fig. S15. Hash signs (#) denote samples before these points were below the limit of detection for IgG1-glycosylation analyses. Vertical dotted lines in (A) and (B) indicate the time of ICU admission and ICU discharge (black) or death (red), whereas dotted horizontal lines in (B) and (D) indicate IL-6 and CRP detection limits.

We then asked how these afucosylated anti-SARS-CoV-2 antibodies might contribute to the strong inflammatory response observed in ARDS patients. Alveolar macrophages are front-line scavengers in the lung and express FcγRIIIa, the major myeloid sensory receptor for afucosylated IgG. Thus, we examined their potential to stimulate the production of the pro-inflammatory cytokine interleukin-6 (IL-6), the cytokine that is most critical for acute-phase responses in humans (*29*). Afucosylated IgG, together with Toll-like receptor 3 (TLR3) ligand, enhanced IL-6 production from macrophages in vitro, particularly when using afucosylated and highly galactosylated IgG, as is found prominently in the ARDS patients (Fig. 3N and figs. S8 and S13). There was a significant correlation between anti-S IgG1-Fc fucosylation and both plasma IL-6 and C-reactive protein (CRP) concentrations (Fig. 3, O and P). This agrees with our hypothesis that afucosylated anti–SARS-CoV-2 IgG plays a substantial role in COVID-19 pathogenesis. Concentrations of plasma IL-6 and CRP increased around the time when afucosylated anti-S IgG appeared, which suggested a direct causality (Fig. 4, A and B). Plasma D-dimer levels also shared this temporal pattern (fig. S14). Thus, the afucosylated and highly galactosylated anti-S and anti-N IgG in some patients may cause an exaggerated release of proinflammatory cytokines and subsequent systemic inflammation because of their enhanced binding capacity to FcγRIIIa (*24*) on alveolar macrophages. No increase of either IL-6 or CRP was observed in the non-ARDS cases (Fig. 4, C and D, and fig. S15).

## Discussion

Our results show a pattern of afucosylated IgG1 immune responses against membrane-embedded antigens such as surface membrane proteins of alloantigens on blood cells or on enveloped viruses (including attenuated enveloped virus vaccines that often complete their first round of infection). This contrasts with soluble protein antigens and nonenveloped viruses for which immune responses with high levels of IgG1-Fc fucosylation were consistently observed. Although there was afucosylated anti-N IgG in COVID-19 patients, this was no longer the case 1 to 2 weeks after seroconversion.

**Fig. 5:**
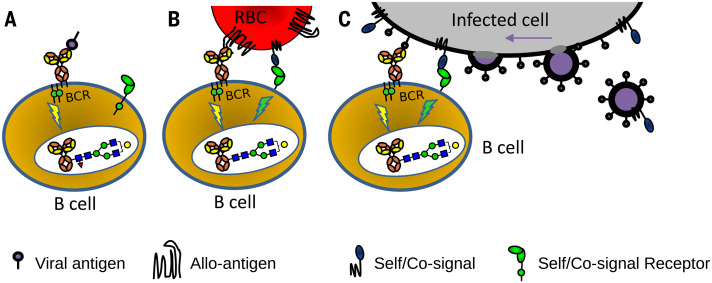
Hypothetical model explaining how different antigen contexts could produce altered immune signaling that gives rise to altered IgG glycosylation. (**A**) Immune responses to soluble protein antigen: B cell receptor (BCR; a membrane Ig) is activated, resulting in the production of normal fucosylated antibodies. (**B**) For immune responses to alloantigens, paternal alloantigens on a red blood cell (RBC) are recognized by the BCR and possibly by other undescribed immune regulatory receptor-ligand pair(s) that provide a signal for recognition of self. (**C**) For immune responses to enveloped viral infection and attenuated viruses, the recognition of enveloped virus–infected cells by B cells would be similar as for the recognition of cellular alloantigens (B). The initial recognition may potentially occur toward enveloped virus–infected cells and/or after viral assembly (far right). The proposed signaling in (B) and (C) causes altered glyco-programming of the B cells, culminating in a distinct IgG response characterized by a low Fc fucosylation (red triangle, fucose) and enhanced ADCC. This model potentially explains both why immune responses to soluble proteins, nonenveloped viruses, and cellular pathogens such as bacteria are different from responses to enveloped viruses (and attenuated viruses). Furthermore, it may explain why immune responses to alloantigens immunologically resemble those of enveloped viral infections.

We hypothesize that antigen-presenting membranes are directly sensed by B cells by combining at least two signals provided by the B cell receptor and undescribed host receptor-ligand pair(s). This two-step mechanism would be essential for the production of long-lasting afucosylated IgG responses and would not be triggered by soluble proteins, internal proteins of enveloped viruses, or nonenveloped viruses (Fig. 5). Alternatively, differential antigen recognition may be more complex and require additional interactions from antigen-presenting cells, T cells, and/or cytokines. This notion is supported by anti-N SARS-CoV-2 responses occurring concomitantly with anti-S responses, which suggests that proximal factors in the lymphoid microenvironment can influence the response.

This work suggests that providing foreign B cell antigens in the context of the host-cell membrane may be necessary but not sufficient to trigger an immune response with high amounts of long-lasting afucosylated IgG (*17*). This translates into varied Fc fucosylation levels between individuals as well as for distinct responses of the same individual against different antigens. The large difference in the number of antigen-specific afucosylated responses observed between patients contributes to the variability of disease severity, as has been shown for neonatal alloimmune cytopenias (*12*, *13*, *17*) and dengue (*15*). Here, we also show its importance for the pathogenesis of COVID-19. Thus, afucosylation may potentially help predict disease trajectories and guide future treatments aimed at minimizing this FcγRIIIa stimulus.

IgG-Fc afucosylation results in potent immune responses. Namely, FcγRIIIa-expressing natural killer (NK) cells, monocytes, and macrophages as well as FcγRIIIb-expressing granulocytes are triggered to destroy target cells. This response may be desirable in some responses, such as against HIV (*16*), and can be achieved with available attenuated enveloped viral vaccine shuttles (*30*) against targets for which vaccine-based approaches have failed. However, this phenomenon can also lead to an undesirable exaggerated response, as is the case for both dengue virus (*15*) and SARS-CoV-2. Attenuated virus vaccine ferrying spike proteins of SARS-CoV are known to produce strong antibody-dependent enhanced responses (*31*) mimicking pathologies in critically ill SARS-CoV-2 patients (*21*). This suggests that subunit protein vaccines may be a safer option, as seen in rat models for SARS-CoV-2 (*32*), unless the vaccine also induces a strong neutralizing effect that can contribute to enhanced protection.

The afucosylation of anti-S IgG may contribute to the exacerbation of COVID-19 in a subset of patients, resulting in ARDS. Thus, although they can be protective, antibodies potentially behave as double-edged swords and may contribute to the observed cytokine storm (*33*). As such, this has direct consequences for the development of improved intravenous Ig (IVIg), convalescent plasma, and vaccine therapies. In addition, the suggested role of afucosylated antibodies in the pathogenesis of SARS-CoV-2 may open up additional opportunities for the treatment of COVID-19. Thus, attempts to generate high-titer Ig treatments should preferably use plasma enriched in fucosylated anti–SARS-CoV-2 antibodies. This may avoid the escalation of symptoms and promote virus neutralization in patients, preferentially before developing afucosylated IgG responses.

## Materials and methods

### Patient samples

Healthy blood donor samples from Sanquin, Amsterdam, the Netherlands, were used to analyze parvovirus B19 (*n* = 22), measles virus (*n* = 24 natural infection, *n* = 21 live-attenuated vaccine), mumps virus (*n* = 24 natural infection, *n* = 21 live-attenuated vaccine), and HBV antibodies (*n* = 17 natural infection, *n* = 17 HBsAg vaccination). Anti-HPA-1a samples have been described elsewhere (*14*). HIV-samples (*n* = 80) from the Amsterdam Cohort Studies on HIV infection and AIDS (ACS) were used to analyze HIV-specific antibody glycosylation. Peripheral blood samples used to purify CMV-specific antibodies (*n* = 102) were from same cohort as used for HPA-1a collected by the Finnish Red Cross Blood service, Platelet Immunology Laboratory, Helsinki, Finland and the HIV cohort described above. SARS-CoV-2 patient samples from ICU patients from the Amsterdam UMC COVID study group were included, as well as Sanquin blood donors found seropositive for SARS-CoV-2, and mild longitudinal samples from hospital workers monitored after potential exposure (*26*). A summary of the patients demographics is found in table S1, and detailed patient treatments of the ARDS COVID-19 patients in table S2 (all requiring ventilation). The ACS have been conducted in accordance with the ethical principles set out in the declaration of Helsinki and all participants provided written informed consent. The study was approved by the Academic Medical Center institutional Medical Ethics Committee of the University of Amsterdam.

### Purification of CMV-specific antibodies from sera

CMV-specific antibodies were purified using antigen-coated plates (Serion ELISA classic, Cytomegalovirus IgG, Würzburg, Germany). Sera (20 μl) were diluted in specimen diluent (80 μl) from the kit and then incubated in the plates for 1 hour at 37°C. Positive and negative controls from the kit and CMV-negative patients samples were used as controls. The plates were washed three times with wash buffer (300 μl) from the kit, twice with phosphate buffered saline (PBS) (300 μl), and twice with deionized water (300 μl). The bound antibodies were then eluted with 100 μl of 100 mM formic acid. No IgG was found in eluates from blank wells and CMV-negative patient samples.

### Purification of measles virus– and mumps virus–specific antibodies from sera

Ag-specific antibodies were purified using antigen-coated plates (Serion ELISA classic, Measles IgG and Mumps IgG, Würzburg, Germany). Sera (20 μl) were diluted in specimen diluent (80 μl) from the kit and then incubated in the plates for 1 hour at 37°C. Positive and negative controls from the kit were used. The plates were washed three times with wash buffer (300 μl) from the kit, twice with PBS (300 μl), and twice 50 mM ammonium bicarbonate (300 μl). The bound antibodies were then eluted with 100 μl of 100 mM formic acid. IgG was found in the eluates of positive controls, and no IgG was found in eluates from blank wells and negative control samples.

### Purification of HBV-specific antibodies from sera

To isolate HBsAg specific antibodies from patients after infection and vaccination, HB antigen-coated plates (ETI-AB-AUK-3, Diasorin, Schiphol-Rijk, the Netherlands) were used. Sera (20 μl) were diluted in specimen diluent (80 μl) from the kit and then incubated in the plates for 1 hour at room temperature (RT) with shaking. HBV-naive and HBV-resolved samples from Sanquin, Amsterdam, the Netherlands were used as controls. Washing and elution of specific antibodies was performed as described above for CMV-specific antibodies.

### Purification of HIV-specific antibodies from sera

HIV-specific antibodies were isolated using HIV antigen-coated plates (Murex HIV1.2.0 kit 9E25-01, Diasorin, Schiphol-Rijk, the Netherlands). Sera were diluted (50 μl) were diluted in specimen diluent (50 μl) from the kit and then incubated in the plates for 1 hour at room temperature (RT) with shaking. As a positive control, anti-HIV gp120 monoclonal was used (IgG1 b12; 100 μg of purified antibody in PBS at 1 mg/ml; NIH Aids Reagent Program, La Jolla, CA, USA). Washing and elution of specific antibodies was performed as described above for CMV-specific antibodies.

### Purification of parvovirus B19-specific antibodies from sera

Parvovirus B19-specific antibodies were isolated using parvovirus B19 antigen-coated plates (Abcam1788650- Anti-Parvovirus B19 IgG ELISA, Cambridge, UK). Sera (20 μl) were diluted in specimen diluent (80 μl) from the kit and then incubated in the plates for 1 hour at room temperature (RT) with shaking. Positive and negative controls from the kit were used as controls. Washing and eluting specific antibodies was performed as described above for CMV-specific antibodies.

### Purification of anti-N and anti-S specific antibodies from plasma

SARS-Cov-2–specific antibodies were purified using antigen-coated plates (NUNC, Roskilde, Denmark). Plates were coated overnight at 4°C with recombinant trimerized spike protein produced as described recently (*34*) or N protein [GenBank: MN908947, produced in HEK cells with a HAVT20 leader peptide, 10x His tag, and a BirA tag (*24*)] in PBS (5 μg/ml and 1 μg/ml, respectively). Plates were washed three times with PBS (250 μl) supplemented with 0.05% TWEEN 20 (PBS-T). Plasma (20 μl) was diluted in PBS-T (180 μl) and then incubated for 1 hour at room temperature (RT) with shaking. Sera dating pre COVID-19 pandemic were used as negative controls. The plates were washed three times with PBS-T (250 μl), twice with PBS (250 μl), and twice with 250 μl ammonium bicarbonate (50 mM). The bound antibodies were then eluted with 100 mM formic acid (200 μl).

### Purification of total IgG from sera

Total IgG1 antibodies were captured from 2 μl of serum using Protein G Sepharose 4 Fast Flow beads (GE Healthcare, Uppsala, Sweden) in a 96-well filter plate (Millipore Multiscreen, Amsterdam, the Netherlands) as previously described (*12*) or by using Protein G cartridges on the AssayMAP Bravo (Agilent Technologies, Santa Clara, USA). Briefly, 1 μl of serum diluted in PBS was applied to the cartridges, followed by washes with PBS and LC-MS pure water. IgG antibodies were then eluted with 1% formic acid.

### Mass spectrometric IgG-Fc glycosylation analysis

Eluates containing either antigen-specific antibodies or total IgG were collected in V-bottom plates and dried by vacuum centrifugation for 2.5 hours at 50°C. The HPA1a, CMV, HIV, Parvovirus B19, HBV, and COVID-19 samples were then subjected to proteolytic cleavage using trypsin as described before (*12*). The measles and mumps cohort samples were dissolved in a buffer containing 0.4% sodium deoxycholate (SDC), 10 mM TCEP, 40 mM chloroacetamide, and 100 mM TRIS pH 8.5. After a 10-min incubation at 95°C, 250 ng of trypsin in 50 mM ammonium bicarbonate was added. The digestion was stopped after an overnight incubation by acidifying to 2% formic acid. Prior to MS injection, SDC precipitates were removed by centrifuging samples at 20,000*g* for 30 min. Analysis of IgG Fc-glycosylation was performed with nanoLC reverse phase (RP)–electrospray (ESI)–MS on an Ultimate 3000 RSLCnano system (Dionex/Thermo Scientific, Breda, the Netherlands) coupled to an amaZon speed ion trap MS (Bruker Daltonics, Bremen, Germany) as described previously (12). Alternatively, the measles, mumps, and COVID-19 cohorts were measured on an ImpactHD quadrupole-time-of-flight MS (Bruker Daltonics) as previously described (*35*). In the current study, we focused on IgG1, without analyzing IgG3 due to its possible interference with IgG2 and IgG4 at the glycopeptide level (*36*). Mass spectrometry results were extracted and evaluated using DataAnalysis software (version 5.0; Bruker Daltonics) for all samples except for the measles, mumps, and COVID-19 cohorts that were analyzed with Skyline software (version 4.2.19107). Data was judged reliable when the sum of the signal intensities of all glycopeptide species (table S3) was higher than negative samples plus 10 times its standard division. Otherwise, the data was excluded (*12*). The total level of glycan traits was calculated as described in table S4.

### Cytokine release assay

Monocytes were isolated from buffy coats and differentiated as previously described (*37*) using M-CSF and IL-10. This results in a phenotype resembling alveolar macrophage-like monocyte-derived macrophages (*37*, *38*). To generated IgG immune complexes, 2 μg/ml of glycoengineered IgG1 (*39*) was coated overnight in PBS on a 96-well high-affinity plate (Nunc; Roskilde; Denmark). Macrophages (50,000/well) were stimulated in pre-coated plates as described in legend in combination with 20 μg/ml of poly(I:C) (Sigma-Aldrich). To measure IL-6 production, supernatants were harvested after 24 hours of stimulation. IL-6 was then detected using an IL-6 enzyme-linked immunosorbent assay (ELISA) kit according to the manufacturer’s instructions (U-CyTech Biosciences). Both coating and detection antibodies were diluted 1:200.

### Meso Scale Discovery multiplex assay

V-PLEX Custom Human Cytokine10-plex kit was purchased from Meso Scale Discovery (MSD). The lyophilized cocktail mix calibrators were reconstituted in provided assay diluents respectively. Plasma and sera (10 μl) were diluted in 40 μl MSD Sample Diluent for IL-6 measurement. The assay was performed according to the manufacturer’s instructions with an overnight incubation of the diluted samples and standards at 4°C. The electrochemiluminescence signal (ECL) was detected by MESO QuickPlex SQ 120 plate reader (MSD) and analyzed with Discovery Workbench Software (v4.0, MSD).

### Anti–SARS-CoV2 antibody levels

Antibody levels were quantified by ELISA. Briefly, samples were tested at 100- to 1200-fold dilutions in PBS supplemented with 0.1% polysorbate-20 and 0.3% gelatin (PTG) in microtiter plates coated with S or N-protein and incubated for 1 hour at RT. Both proteins were produced as previously described (*26*). After washing, 0.5 μg/ml of HRP-conjugated anti-human IgG (MH16-1, Sanquin) was added in PTG and incubated for 1 hour at RT. Following enzymatic conversion of TMB substrate, absorbance was measured at 450 nm and 540 nm. Antibody binding was evaluated by comparison to a reference plasma pool of convalescent COVID-19 patients set at 100 AU/ml.

### Statistical analysis

Statistical analyses were performed using GraphPad Prism (version 8.0.2) for Windows (GraphPad Software, La Jolla, CA; www.graphpad.com). To analyze whether Fc-fucosylation for total and antigen-specific IgG differs between the tested cohorts, statistical analysis was performed using two-way analysis of variance (ANOVA) and paired *t* tests as specified for the individual cohorts. The same tests were used for comparing cytokine release from stimulated macrophages. To investigate whether Fc-fucosylation profiles of two specific antibodies in the same individual were correlated, statistical analysis was performed using a Pearson’s correlation. A Pearson’s correlation was also used to test the correlation between Fc-fucosylation of anti-S and anti-N IgG. To test correlations between cytokine release and IgG Fc-fucosylation, as well as between the degree of anti-S Fc fucosylation and CRP levels, a Spearman’s correlation was performed. Only statistically significant differences are shown; *P < 0.05, **P < 0.01, ***P < 0.001, ****P < 0.0001.

## Supplementary Materials

science.sciencemag.org/content/371/6532/eabc8378/suppl/DC1 Figs. S1 to S15 Tables S1 to S4 Amsterdam UMC COVID-19 biobank study group MDAR Reproducibility Checklist

## Appendix

View/request a protocol for this paper from *Bio-protocol*.

## References

[1] M. H. C. Biermann, G. Griffante, M. J. Podolska, S. Boeltz, J. Stürmer, L. E. Muñoz, R. Bilyy, M. Herrmann, Sweet but dangerous—The role of immunoglobulin G glycosylation in autoimmunity and inflammation. Lupus 25, 934–942 (2016). 10.1177/0961203316640368 27252272

[2] R. Jefferis, Glycosylation as a strategy to improve antibody-based therapeutics. Nat. Rev. Drug Discov. 8, 226–234 (2009). 10.1038/nrd2804 19247305

[3] G. Vidarsson, G. Dekkers, T. Rispens, IgG subclasses and allotypes: From structure to effector functions. Front. Immunol. 5, 520 (2014). 10.3389/fimmu.2014.00520 25368619 PMC4202688

[4] G. Dekkers, T. Rispens, G. Vidarsson, Novel concepts of altered immunoglobulin G galactosylation in autoimmune diseases. Front. Immunol. 9, 553 (2018). 10.3389/fimmu.2018.00553 29616041 PMC5867308

[5] R. L. Shields, J. Lai, R. Keck, L. Y. O’Connell, K. Hong, Y. G. Meng, S. H. A. Weikert, L. G. Presta, Lack of fucose on human IgG1 N-linked oligosaccharide improves binding to human Fcgamma RIII and antibody-dependent cellular toxicity. J. Biol. Chem. 277, 26733–26740 (2002). 10.1074/jbc.M202069200 11986321

[6] C. Ferrara, S. Grau, C. Jäger, P. Sondermann, P. Brünker, I. Waldhauer, M. Hennig, A. Ruf, A. C. Rufer, M. Stihle, P. Umaña, J. Benz, Unique carbohydrate-carbohydrate interactions are required for high affinity binding between FcγRIII and antibodies lacking core fucose. Proc. Natl. Acad. Sci. U.S.A. 108, 12669–12674 (2011). 10.1073/pnas.1108455108 21768335 PMC3150898

[7] J. M. Reichert, Antibodies to watch in 2016. MAbs 8, 197–204 (2016). 10.1080/19420862.2015.1125583 26651519 PMC4966626

[8] M. P. Baković, M. H. J. Selman, M. Hoffmann, I. Rudan, H. Campbell, A. M. Deelder, G. Lauc, M. Wuhrer, High-throughput IgG Fc N-glycosylation profiling by mass spectrometry of glycopeptides. J. Proteome Res. 12, 821–831 (2013). 10.1021/pr300887z 23298168

[9] N. de Haan, K. R. Reiding, G. Driessen, M. van der Burg, M. Wuhrer, Changes in healthy human IgG Fc-glycosylation after birth and during early childhood. J. Proteome Res. 15, 1853–1861 (2016). 10.1021/acs.jproteome.6b00038 27161864

[10] X. Yu, Y. Wang, J. Kristic, J. Dong, X. Chu, S. Ge, H. Wang, H. Fang, Q. Gao, D. Liu, Z. Zhao, H. Peng, M. Pucic Bakovic, L. Wu, M. Song, I. Rudan, H. Campbell, G. Lauc, W. Wang, Profiling IgG N-glycans as potential biomarker of chronological and biological ages: A community-based study in a Han Chinese population. Medicine (Baltimore) 95, e4112 (2016). 10.1097/MD.0000000000004112 27428197 PMC4956791

[11] M. Wuhrer, L. Porcelijn, R. Kapur, C. A. M. Koeleman, A. Deelder, M. de Haas, G. Vidarsson, Regulated glycosylation patterns of IgG during alloimmune responses against human platelet antigens. J. Proteome Res. 8, 450–456 (2009). 10.1021/pr800651j 18942870

[12] R. Kapur, I. Kustiawan, A. Vestrheim, C. A. M. M. Koeleman, R. Visser, H. K. Einarsdottir, L. Porcelijn, D. Jackson, B. Kumpel, A. M. Deelder, D. Blank, B. Skogen, M. K. Killie, T. E. Michaelsen, M. de Haas, T. Rispens, C. E. van der Schoot, M. Wuhrer, G. Vidarsson, A prominent lack of IgG1-Fc fucosylation of platelet alloantibodies in pregnancy. Blood 123, 471–480 (2014). 10.1182/blood-2013-09-527978 24243971 PMC3901064

[13] R. Kapur, L. Della Valle, M. Sonneveld, A. Hipgrave Ederveen, R. Visser, P. Ligthart, M. de Haas, M. Wuhrer, C. E. van der Schoot, G. Vidarsson, Low anti-RhD IgG-Fc-fucosylation in pregnancy: A new variable predicting severity in haemolytic disease of the fetus and newborn. Br. J. Haematol. 166, 936–945 (2014). 10.1111/bjh.12965 24909983 PMC4282073

[14] M. E. Sonneveld, S. Natunen, S. Sainio, C. A. M. Koeleman, S. Holst, G. Dekkers, J. Koelewijn, J. Partanen, C. E. van der Schoot, M. Wuhrer, G. Vidarsson, Glycosylation pattern of anti-platelet IgG is stable during pregnancy and predicts clinical outcome in alloimmune thrombocytopenia. Br. J. Haematol. 174, 310–320 (2016). 10.1111/bjh.14053 27017954

[15] T. T. Wang, J. Sewatanon, M. J. Memoli, J. Wrammert, S. Bournazos, S. K. Bhaumik, B. A. Pinsky, K. Chokephaibulkit, N. Onlamoon, K. Pattanapanyasat, J. K. Taubenberger, R. Ahmed, J. V. Ravetch, IgG antibodies to dengue enhanced for FcγRIIIA binding determine disease severity. Science 355, 395–398 (2017). 10.1126/science.aai8128 28126818 PMC5557095

[16] M. E. Ackerman, M. Crispin, X. Yu, K. Baruah, A. W. Boesch, D. J. Harvey, A.-S. S. Dugast, E. L. Heizen, A. Ercan, I. Choi, H. Streeck, P. A. Nigrovic, C. Bailey-Kellogg, C. Scanlan, G. Alter, Natural variation in Fc glycosylation of HIV-specific antibodies impacts antiviral activity. J. Clin. Invest. 123, 2183–2192 (2013). 10.1172/JCI65708 23563315 PMC3637034

[17] M. E. Sonneveld, J. Koelewijn, M. de Haas, J. Admiraal, R. Plomp, C. A. M. Koeleman, A. L. Hipgrave Ederveen, P. Ligthart, M. Wuhrer, C. E. van der Schoot, G. Vidarsson, Antigen specificity determines anti-red blood cell IgG-Fc alloantibody glycosylation and thereby severity of haemolytic disease of the fetus and newborn. Br. J. Haematol. 176, 651–660 (2017). 10.1111/bjh.14438 27891581

[18] M. H. J. Selman, S. E. de Jong, D. Soonawala, F. P. Kroon, A. A. Adegnika, A. M. Deelder, C. H. Hokke, M. Yazdanbakhsh, M. Wuhrer, Changes in antigen-specific IgG1 Fc N-glycosylation upon influenza and tetanus vaccination. Mol. Cell. Proteomics 11, 014563 (2012). 10.1074/mcp.M111.014563 22184099 PMC3322571

[19] J. Krištić, F. Vučković, C. Menni, L. Klarić, T. Keser, I. Beceheli, M. Pučić-Baković, M. Novokmet, M. Mangino, K. Thaqi, P. Rudan, N. Novokmet, J. Sarac, S. Missoni, I. Kolčić, O. Polašek, I. Rudan, H. Campbell, C. Hayward, Y. Aulchenko, A. Valdes, J. F. Wilson, O. Gornik, D. Primorac, V. Zoldoš, T. Spector, G. Lauc, Glycans are a novel biomarker of chronological and biological ages. J. Gerontol. A Biol. Sci. Med. Sci. 69, 779–789 (2014). 10.1093/gerona/glt190 24325898 PMC4049143

[20] L. Bouadma, F. X. Lescure, J. C. Lucet, Y. Yazdanpanah, J. F. Timsit, Severe SARS-CoV-2 infections: Practical considerations and management strategy for intensivists. Intensive Care Med. 46, 579–582 (2020). 10.1007/s00134-020-05967-x 32103284 PMC7079839

[21] C. Huang, Y. Wang, X. Li, L. Ren, J. Zhao, Y. Hu, L. Zhang, G. Fan, J. Xu, X. Gu, Z. Cheng, T. Yu, J. Xia, Y. Wei, W. Wu, X. Xie, W. Yin, H. Li, M. Liu, Y. Xiao, H. Gao, L. Guo, J. Xie, G. Wang, R. Jiang, Z. Gao, Q. Jin, J. Wang, B. Cao, Clinical features of patients infected with 2019 novel coronavirus in Wuhan, China. Lancet 395, 497–506 (2020). 10.1016/S0140-6736(20)30183-5 31986264 PMC7159299

[22] C. Ceraolo, F. M. Giorgi, Genomic variance of the 2019-nCoV coronavirus. J. Med. Virol. 92, 522–528 (2020). 10.1002/jmv.25700 32027036 PMC7166773

[23] T. T. Wang, J. Maamary, G. S. Tan, S. Bournazos, C. W. Davis, F. Krammer, S. J. Schlesinger, P. Palese, R. Ahmed, J. V. Ravetch, Anti-HA Glycoforms Drive B Cell Affinity Selection and Determine Influenza Vaccine Efficacy. Cell 162, 160–169 (2015). 10.1016/j.cell.2015.06.026 26140596 PMC4594835

[24] G. Dekkers, L. Treffers, R. Plomp, A. E. H. Bentlage, M. de Boer, C. A. M. Koeleman, S. N. Lissenberg-Thunnissen, R. Visser, M. Brouwer, J. Y. Mok, H. Matlung, T. K. van den Berg, W. J. E. van Esch, T. W. Kuijpers, D. Wouters, T. Rispens, M. Wuhrer, G. Vidarsson, Decoding the human immunoglobulin G-glycan repertoire reveals a spectrum of Fc-receptor- and complement-mediated-effector activities. Front. Immunol. 8, 877 (2017). 10.3389/fimmu.2017.00877 28824618 PMC5539844

[25] A. R. Temming, S. W. de Taeye, E. L. de Graaf, L. A. de Neef, G. Dekkers, C. W. Bruggeman, J. Koers, P. Ligthart, S. Q. Nagelkerke, J. C. Zimring, T. W. Kuijpers, M. Wuhrer, T. Rispens, G. Vidarsson, Functional attributes of antibodies, effector cells, and target cells affecting NK cell-mediated antibody-dependent cellular cytotoxicity. J. Immunol. 203, 3126–3135 (2019). 10.4049/jimmunol.1900985 31748349

[26] E. H. Vogelzang, F. C. Loeff, N. I. L. Derksen, S. Kruithof, P. Ooijevaar-de Heer, G. van Mierlo, F. Linty, J. Y. Mok, W. van Esch, S. de Bruin, A. P. J. Vlaar, B. Seppen, M. Leeuw, A. J. G. van Oudheusden, A. G. M. Buiting, K. K. Jim, H. Vrielink, F. Swaneveld, G. Vidarsson, C. E. van der Schoot, P. C. Wever, W. Li, F. van Kuppeveld, J.-L. Murk, B. J. Bosch, G.-J. J. Wolbink, T. Rispens, Amsterdam University Medical Center COVID-19 Biobank Study Group, Development of a SARS-CoV-2 total antibody assay and the dynamics of antibody response over time in hospitalized and nonhospitalized patients with COVID-19. J. Immunol. 205, 3491–3499 (2020). 10.4049/jimmunol.2000767 33127820

[27] R. Kapur, L. Della Valle, O. J. H. M. Verhagen, A. Hipgrave Ederveen, P. Ligthart, M. de Haas, B. Kumpel, M. Wuhrer, C. E. van der Schoot, G. Vidarsson, Prophylactic anti-D preparations display variable decreases in Fc-fucosylation of anti-D. Transfusion 55, 553–562 (2015). 10.1111/trf.12880 25234110

[28] N. L. Bernasconi, E. Traggiai, A. Lanzavecchia, Maintenance of serological memory by polyclonal activation of human memory B cells. Science 298, 2199–2202 (2002). 10.1126/science.1076071 12481138

[29] M. W. N. Nijsten, E. R. de Groot, H. J. ten Duis, H. J. Klasen, C. E. Hack, L. A. Aarden, Serum levels of interleukin-6 and acute phase responses. Lancet 2, 921 (1987). 10.1016/S0140-6736(87)91413-9 2889120

[30] A. Volz, G. Sutter, Modified Vaccinia Virus Ankara: History, Value in basic research, and current perspectives for vaccine development. Adv. Virus Res. 97, 187–243 (2017). 10.1016/bs.aivir.2016.07.001 28057259 PMC7112317

[31] L. Liu, Q. Wei, Q. Lin, J. Fang, H. Wang, H. Kwok, H. Tang, K. Nishiura, J. Peng, Z. Tan, T. Wu, K. W. Cheung, K. H. Chan, X. Alvarez, C. Qin, A. Lackner, S. Perlman, K. Y. Yuen, Z. Chen, Anti-spike IgG causes severe acute lung injury by skewing macrophage responses during acute SARS-CoV infection. JCI Insight 4, e123158 (2019). 10.1172/jci.insight.123158 30830861 PMC6478436

[32] B. Quinlan, H. Mou, L. Zhang, Y. Guo, W. He, A. Ojha, M. Parcells, G. Luo, W. Li, G. Zhong, H. Choe, M. Farzan, bioRxiv 036418 [Preprint]. 12 April 2020. . 10.1101/2020.04.10.036418

[33] Q. Ye, B. Wang, J. Mao, The pathogenesis and treatment of the ‘Cytokine Storm’ in COVID-19. J. Infect. 80, 607–613 (2020). 10.1016/j.jinf.2020.03.037 32283152 PMC7194613

[34] P. J. M. Brouwer, T. G. Caniels, K. van der Straten, J. L. Snitselaar, Y. Aldon, S. Bangaru, J. L. Torres, N. M. A. Okba, M. Claireaux, G. Kerster, A. E. H. Bentlage, M. M. van Haaren, D. Guerra, J. A. Burger, E. E. Schermer, K. D. Verheul, N. van der Velde, A. van der Kooi, J. van Schooten, M. J. van Breemen, T. P. L. Bijl, K. Sliepen, A. Aartse, R. Derking, I. Bontjer, N. A. Kootstra, W. J. Wiersinga, G. Vidarsson, B. L. Haagmans, A. B. Ward, G. J. de Bree, R. W. Sanders, M. J. van Gils, Potent neutralizing antibodies from COVID-19 patients define multiple targets of vulnerability. Science 369, 643–650 (2020). 10.1126/science.abc5902 32540902 PMC7299281

[35] D. Falck, B. C. Jansen, N. de Haan, M. Wuhrer, High-throughput analysis of IgG Fc glycopeptides by LC-MS. Methods Mol. Biol. 1503, 31–47 (2017). 10.1007/978-1-4939-6493-2_4 27743357

[36] M. Wuhrer, J. C. Stam, F. E. van de Geijn, C. A. M. Koeleman, C. T. Verrips, R. J. E. M. Dolhain, C. H. Hokke, A. M. Deelder, Glycosylation profiling of immunoglobulin G (IgG) subclasses from human serum. Proteomics 7, 4070–4081 (2007). 10.1002/pmic.200700289 17994628

[37] W. Hoepel, H.-J. Chen, S. Allahverdiyeva, X. Manz, J. Aman, A. U. C.-19 Biobank, P. Bonta, P. Brouwer, S. de Taeye, T. Caniels, K. van der Straten, K. Golebski, G. Griffith, R. Jonkers, M. Larsen, F. Linty, A. Neele, J. Nouta, F. van Baarle, C. van Drunen, A. Vlaar, G. de Bree, R. Sanders, L. Willemsen, M. Wuhrer, H. J. Bogaard, M. van Gils, G. Vidarsson, M. de Winther, J. den Dunnen, Anti-SARS-CoV-2 IgG from severely ill COVID-19 patients promotes macrophage hyper-inflammatory responses. bioRxiv 190140 [Preprint] 13 July 2020. . 10.1101/2020.07.13.190140

[38] H. J. Chen, A. Y. F. Li Yim, G. R. Griffith, W. J. de Jonge, M. M. A. M. Mannens, E. Ferrero, P. Henneman, M. P. J. de Winther, Meta-analysis of in vitro-differentiated macrophages identifies transcriptomic signatures that classify disease macrophages in vivo. Front. Immunol. 10, 2887 (2019). 10.3389/fimmu.2019.02887 31921150 PMC6917623

[39] G. Dekkers, R. Plomp, C. A. M. Koeleman, R. Visser, H. H. von Horsten, V. Sandig, T. Rispens, M. Wuhrer, G. Vidarsson, Multi-level glyco-engineering techniques to generate IgG with defined Fc-glycans. Sci. Rep. 6, 36964 (2016). 10.1038/srep36964 27872474 PMC5131652

